# An easy and efficient permeabilization protocol for in vivo enzyme activity assays in cyanobacteria

**DOI:** 10.1186/s12934-016-0587-3

**Published:** 2016-11-08

**Authors:** Randi Engelberth Rasmussen, Simon Matthé Erstad, Erick Miguel Ramos-Martinez, Lorenzo Fimognari, Alice Jara De Porcellinis, Yumiko Sakuragi

**Affiliations:** Department of Plant and Environmental Sciences, Copenhagen Plant Science Center, University of Copenhagen, Frederiksberg, Denmark

**Keywords:** B-PER, Permeabilization, Cyanobacteria, Glucose-6-phosphate dehydrogenase, Rubisco, SYTOX Green, Enzyme activities

## Abstract

**Background:**

Cyanobacteria are photosynthetic bacteria that thrive in diverse ecosystems and play major roles in the global carbon cycle. The abilities of cyanobacteria to fix atmospheric CO_2_ and to allocate the fixed carbons to chemicals and biofuels have attracted growing attentions as sustainable microbial cell factories. Better understanding of the activities of enzymes involved in the central carbon metabolism would lead to increasing product yields. Currently cell-free lysates are the most widely used method for determination of intracellular enzyme activities. However, due to thick cell walls, lysis of cyanobacterial cells is inefficient and often laborious. In some cases radioisotope-labeled substrates can be fed directly to intact cells; however, label-free assays are often favored due to safety and practical reasons.

**Results:**

Here we show an easy and highly efficient method for permeabilization of the cyanobacteria *Synechococcus* sp. PCC 7002 and *Synechocystis* sp. PCC 6803, and determination of two intracellular enzymes, ribulose-1,5-bisphosphate carboxylase/decarboxylase (Rubisco) and glucose-6-phosphate dehydrogenase (G6PDH), that play pivotal roles in the central carbon metabolism in cyanobacteria. Incubation of the cyanobacterial cells in the commercially available B-PER reagent for 10 min permeabilized the cells, as confirmed by the SYTOX Green staining. There was no significant change in the cell shape and no major loss of intracellular proteins was observed during the treatment. When used directly in the assays, the permeabilized cells exhibited the enzyme activities that are comparable or even higher than those detected for cell-free lysates. Moreover, the permeabilized cells could be stored at −20 °C without losing the enzyme activities. The permeabilization process and subsequent activity assays were successfully adapted to the 96-well plate system.

**Conclusions:**

An easy, efficient and scalable permeabilization protocol was established for cyanobacteria. The permeabilized cells can be directly applied for measurement of G6PDH and Rubisco activities without using radioisotopes and the protocol may be readily adapted to studies of other cyanobacterial species and other intracellular enzymes. The permeabilization and enzyme assays can be performed in 96-well plates in a high-throughput manner.

## Background

Cyanobacteria are photosynthetic bacteria that thrive in diverse ecosystems [1]. They are major players in the global carbon cycle [2], and the metabolic plasticity of cyanobacteria has been a subject of extensive studies over decades. Moreover, growing interests for a sustainable bioeconomy has driven the recent advances in metabolic engineering of cyanobacteria for production of chemicals and biofuels from CO_2_ and light. To date, over 17 non-native biosynthetic pathways have been introduced for production of a wide range of fuel components, platform chemicals, and high-value compounds [3].

Cyanobacteria fix CO_2_ in the light mainly through the Calvin-Benson-Bassham cycle (hereafter the CBB cycle) as basis for growth and generation of storage carbohydrates (e.g., glycogen) [1]. In the dark, these storage carbohydrates are broken down to generate phosphorylated hexose intermediates, reducing equivalents and ATP through the oxidative pentose phosphate pathway (OPPP), the Embden–Meyerhof–Parnas pathway (glycolysis) and the Entner-Doudrofff pathway [4–7]. In addition, a functional phosphoketolase pathway has been reported in engineered strains of *Synechocystis* sp. PCC 6803 (hereafter *Synechocystis* 6803) [8]. Several enzymes in these pathways have been overexpressed, which has led to positive effects with respect to growth and bioproduct synthesis [1]. For example, overexpression of Rubisco, the enzyme responsible for the CO_2_ fixation in the CBB cycle, has been shown to enhance the yields of isobutyraldehydde in *Synechococcus* sp. PCC 7942 [9]. Interestingly, the rate of oxygen evolution was unaltered, which has led to the suggestion that the overexpression of Rubisco leads to more efficient utilization of cellular reductants [9]. G6PDH is the enzyme responsible for the first committing step in OPPP and generates NADPH upon oxidation of glucose-6-phosphate (G6P). It has been shown that overexpression of G6PDH in an ethanol-producing strain of *Synechocystis* 6803 led to an enhanced yield of ethanol, as a result of an increased NADPH pool [10].

As the central carbon metabolic enzymes, the synthesis and activities of Rubisco and G6PDH are tightly regulated. Under oxidizing conditions, the activity of G6PDH is stimulated in a manner dependent on thioredoxins [11–16], whereas the synthesis, the activity, the proteolytic stability of Rubisco have been reported to diminish in part due to the oxidation of cysteine thiol groups [17–20]. The G6PDH activity is inactivated by NADPH and ATP and repressed by ribulose-1,5-bisphosphate, the acceptor for CO_2_ in the CBB cycle [15, 21–24]. Post-translational modifications (phosphorylation and acetylation) of G6PDH have been reported to modulate its activity in plants and humans [20, 21], while the occurrence and the biological functions of these modifications in cyanobacteria are yet to be studied in details [25]. As for Rubisco, transcriptional regulation [26], chaperon-mediated assembly of the holoenzyme [27], encapsulation of the holoenzyme into micro compartments (i.e., carboxysomes) together with carbonic anhydrases [28, 29], and activation of the catalytic sites by both covalent and non-covalent likages [30] are among the regulatory mechanisms that have been previously reported.

Despite the importance of Rubisco and G6PDH in the cyanobacterial central carbon metabolism and apparently also in redox homeostasis, our knowledge about how different environmental stimuli, genetic manipulation, and introduction of heterologous pathways impact the in vivo activities of these enzymes is far from complete. Cell-free lysates are the most widely used cell materials for measurements of intracellular metabolic enzyme activities. However, because cyanobacteria possess a thick peptidoglycan layer [31–33], preparation of cell-free lysates from cyanobacterial cells is often inefficient and/or requires application of mechanical disruption instruments (e.g., ultrasonication and French pressing), which are not practical when tens of samples are processed simultaneously. Moreover, cell-free lysates are limited to in vitro assays that take an enzyme out of its native milieu. Alternatively, whole cells may be used directly by feeding radioisotope-labeled CO_2_ (or bicarbonate in the presence of a carbonic anhydrase) as a substrate for Rubisco (an example has been described in [31]) or ^13^C-labeled CO_2_ or glucose for metabolite flux analyses (examples have been described in [5, 7, 34]). However, feeding of the whole cells with substrates is only possible if they are membrane permeable or actively taken up by the cells. Moreover, concerning radio-labeled substrates, an increasing number of laboratories are opting to move way from radioisotopes due to safety reasons.

Permeabilization offers convenient solutions to these problems. Thus far, organic solvents (toluene, a toluene-methanol mixture), detergents (Triton-X100, alkyltrimethylammonium bromide) and combination thereof with or without lysozyme have been used to permeabilize cyanobacterial cells for determination of nitrate and nitrite reductases in *Synechococcus* sp. PCC 6301 [35], activity assays of enzymes involved in the glycogen biosynthesis in *Anabaena* sp. PCC 7120 [36] and Rubisco in *Anabaena variabilis* and *Synechococcus* sp. PCC 7942 [37–39]. Although these studies demonstrate the utility of permeabilized cells, they provided limited information concerning the efficiency of permeabilization, stability of analyzed enzyme activities during the course of storage, and possible loss of endogenous proteins during the permeabilization treatments.

Here we report preparation and use of permeabilized cyanobacterial cells for in situ activity assays for G6PDH and Rubisco in two cyanobacterial species, *Synechococcus* sp. PCC 7002 (hereafter *Synechococcus* 7002) and *Synechocystis* 6803, that are widely used in research. The method utilizes a commercially available reagent, B-PER (Thermo Fischer Scientific Inc), consisting of non-disclosed non-ionic detergents in 20 mM Tris–HCl, pH 7.5. It is used for a range of bacteria, archaea, insect cells, and mammalian cells, and has been successfully used for mild solubilization of the outer cell layer for recovery of functional proteins [40–47]. We found that a B-PER treatment did not solubilize the cyanobacterial cells, instead it permeabilized the cells. Using the yellow fluorescent protein (YFP) as a reporter, it was confirmed that the permeabilization does not cause a significant loss of intracellular proteins during the procedure. The permeabilized cells could be used directly for determination of the G6PDH activity using exogenously supplied substrates and the Rubisco activity using an enzyme-coupled assay. The activities detected for the permeabilized cells were comparable or even higher than those detected for cell-free lysates obtained by ultrasonication. Notably, both the G6PDH and Rubisco activities remained unaltered after a cycle of freezing and thawing, hence the permeabilized cells can be stored in a freezer for later uses. Lastly, the permeabilization and subsequent enzyme activity assays were adapted to a small scale using 96-well plates for high-throughput analyses.

## Methods

### Bacterial strains and culture conditions

Cells of *Synechococcus* 7002 were grown photoautotrophically in liquid medium A^+^ containing 1 g l^−1^ NaNO_3_ [48] and 0.004 g l^−1^ vitamin B_12_ (known as medium A^+^) at 37 °C with a photon flux of 200 µmol m^−2^ s^−1^. Cells of *Synechocystis* 6803 were grown in liquid medium BG11 at 30 °C with a photon flux of 50 µmol m^−2^ s^−1^. The volumes of the cultures were between 10 ml and 1 l, depending on the amount of the cell materials subsequently required. A continuous supply of 3% (v/v) CO_2_ in air was bubbled through the cultures to provide CO_2_ and agitation. Growth was measured by optical density at 730 nm using an Ultrospec 3100 *pro* UV/Visible Spectrophotometer (Amersham Biosciences, UK). When indicated, 200 µl of cultures were aliquoted in each well in 96-well plates and incubated with a designated concentration of H_2_O_2_ or 3-(3′,4′-dichlorophenyl)-1,1-dimethylurea (DCMU) for 2 h at 26 °C with a photon flux of 20 µmol m^−2^ s^−1^, prior to the permeabilization procedure as described below.

### Permeabilization of cyanobacterial cells

**Fig. 1:**
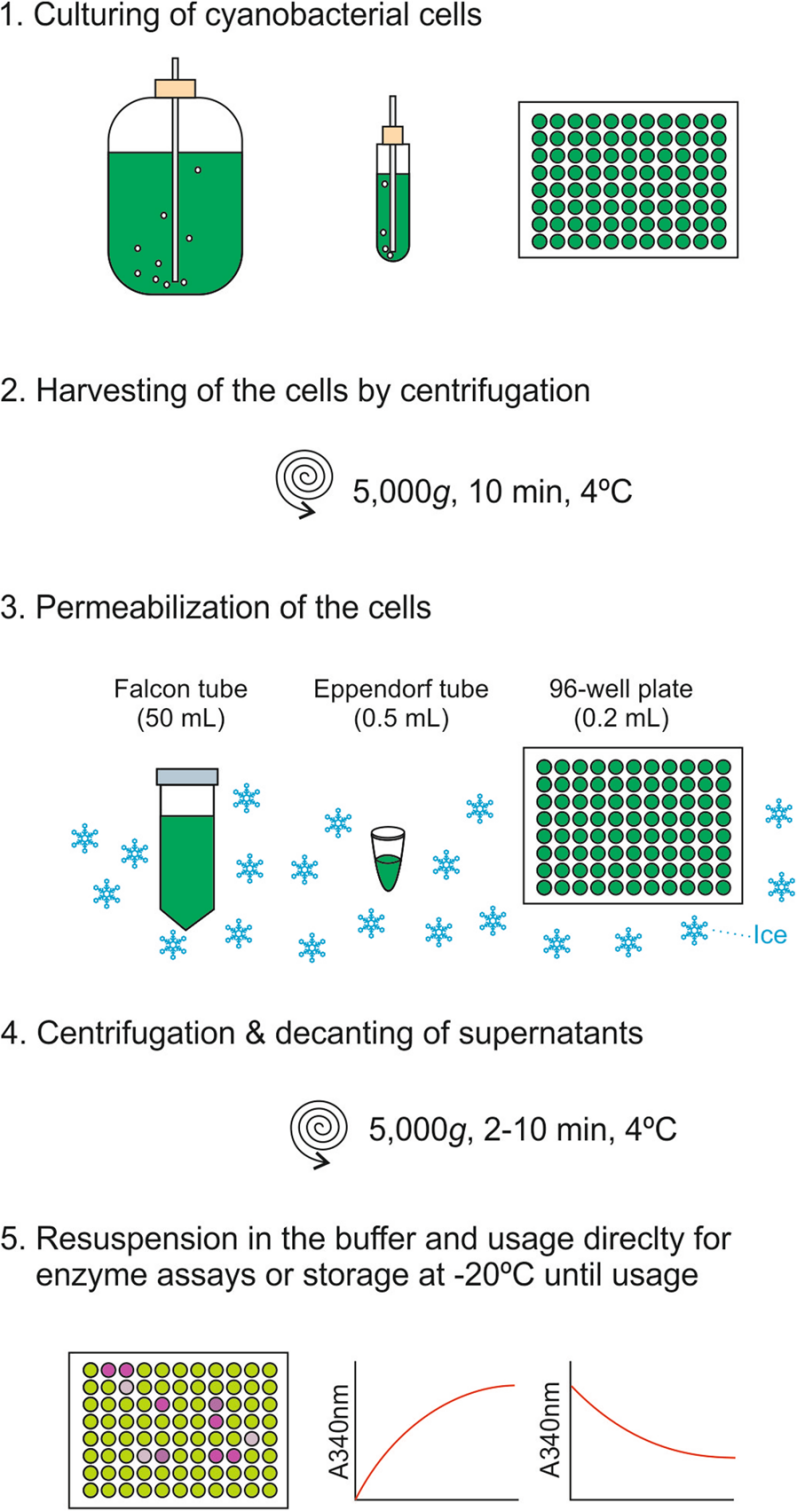
A schematic illustration of the procedure for the permeabilization and enzyme assays. *1.* Cells were grown in volumes ranging from 1 l in a large flask to 0.2 ml in a 96-well plate. *2.* Cells were harvested by centrifugation. *3*. The pellets were resuspended in the B-PER reagent, typically in the volume of 50, 0.5, or 0.1 ml depending on the starting culture volumes, and incubated for 10 min on ice with a gentile agitation. *4.* The permeabilized cells were pelleted by centrifugation and the B-PER reagent was removed. *5*. The permeabilized cell pellets are resuspended in the Tris–HCl buffer and used directly for determination of the enzyme activities and the Chl *a* content

A schematic presentation of the procedure is shown in Fig. 1. Cultures with an optical density value at 730 nm cm^−1^ between 1 and 2, or cultures that have been treated with chemicals, were harvested by centrifugation for 10 min at 5000*g* at 4 °C. The cells were resuspended in one-tenth of the original volume in the B-PER Bacterial Protein Extraction Reagent (Thermo Scientific, U.S.) containing cOmplete ethylenediaminetetraacetic acid (EDTA) free protease inhibitor (Roche, Switzerland) (one tablet in 25 ml of the B-PER reagent), and were shaken gently for 10 min in ice before centrifugation at 5000*g* for 10 min at 4 °C. The pellets were subsequently resuspended in one-fourth the original culture volume of 20 mM Tris–HCl buffer, pH 7.5. The control samples were subjected to the same protocol, except that the B-PER reagent was replaced with the 20 mM Tris–HCl buffer, pH 7.5. The supernatants were discarded unless otherwise stated, and the pellets were resuspended in the buffer, which were used directly for enzyme activity assays and for determination of the chlorophyll *a* (Chl *a*) content. The enzyme activities were normalized by the Chl *a* contents. Alternatively the optical density values at 730 nm cm^−1^ were used to normalize the activities in the small-scale analysis (e.g., in 96-well plates), after having confirmed that the optical density value of cultures did not change significantly during the incubation period.

### Preparation of cell-free extracts

One tablet of cOmplete protease inhibitors was dissolved in 25 ml of Tris–HCl buffer, pH 7.5. Cells harvested from 10 ml of liquid cultures, as described above, were washed and resuspended in the Tris–HCl buffer supplemented with the protease inhibitors to the final volume of 500 µl. The samples were subjected to 20 cycles of 30 s ultrasonication at a frequency of 20 kHz with 10 s intervals at the constant temperature of 4 °C. After centrifugation at 16,000*g* for 1 min, the supernatants were directly used for enzymatic assays and determination of the Chl *a* content.

### Fluorescence microscopy

The cyanobacterial cells were visualized using a TCS SP5 X confocal laser scanning microscopy (Leica Microsystems, Germany) using the Leica Application Suite Advanced Fluorescence software with default settings. Excitation at 573 nm by a super continuum white light laser and emission at 590–640 nm was used to detect intrinsic fluorescence in intact and permeabilized cells, as adapted from [49]. Cells were incubated with SYTOX Green nucleic acid stain (Molecular Probes, US) at the final concentration of 5 µM. The incubated samples were mounted on a slide glass and cells were visualized upon excitation at 488 nm by an argon laser and emission was detected at 515–535 nm [49]. Cell dimensions were measured for cells captured under the bright field by using the Image J software (https://imagej.nih.gov/ij/).

### Immunoblotting

*Synechocystis* 6803 strain expressing YFP [50] were cultivated as described above. Following the B-PER and control treatments, cell pellets and medium supernatants were diluted in the ratio of 3:1 (v/v) in 4× Laemmli sample buffer and loaded in equal proportion on a 12% (v/v) Criterion™ TGX Stain-Free™ Protein Gel, which contains trihalo compounds that can react with tryptophan residues under the UV irradiation and gives rise to fluorescence (BioRad, US). Proteins were subsequently transferred from the gel to a polyvinylidene difluoride membrane. Immunodetection of YFP was carried out using anti-GFP mouse IgG (Roche Applied Science, Germany) according to the manufacturer instructions. Relative signal was quantified using Image Lab™ Software (BioRad, US). The total proteins in the gels were visualized under the UV irradiation according to the manufacturer´s instruction (BioRad, US).

### Determination of enzymatic activities

The G6PDH activity was measured as previously described [15] with minor modifications. Ten microliters of cell-free lysates or permeabilized cells were added to 200 μl of assay buffer comprising 50 mM Tris-Maleate, pH 7.8, 10 mM MgCl_2_, cOmplete protease inhibitor without EDTA (one tablet in 25 ml of the assay buffer), 1 mM NADP^+^, and 10 mM G6P. The rate of NADPH formation at 340 nm was monitored immediately after the addition of the samples for 30 min. For the Rubisco activity assay, 10 µl of cell-free lysate or permeabilized cells were incubated on ice for 10 min in the presence of 10 mM NaHCO_3_ to activate Rubisco [51], and mixed with 240 µl of an assay buffer containing 50 mM Tris–HCl, pH 8.0, 15 mM MgCl_2_, 1 mM EDTA, 5 mM phosphocreatine, 10 mM NaCl, 0.15 mM NADH, 0.25 mM ribulose-1,5-bisphosphate, 10 U glyceraldehyde-3-phosphate dehydrogenase, 10 U 3-phosphoglyceric phosphokinase, and 2.5 U creatine phosphokinase. The rate of NADH oxidation was monitored at 340 nm for 30 min. For all measurements, SpectraMax 190 Microplate Reader (Molecular Devices, US), set at 30 °C, was used. Chl *a* was extracted by vigorously shaking 100 µl of a sample with 900 µl of 96% (v/v) ethanol at 90 °C for 5 min. After centrifugation at 10,000*g* for 10 min, the Chl *a* content was determined spectrophotometrically using an Ultrospec 3100 *pro* UV/Visible Spectrophotometer (Amersham Biosciences, Denmark) as previously described [52].

## Results

### Permeabilization of cyanobacterial cells

Figure 1 shows a schematic representation of the permeabilization procedure. The procedure generates one-fourth the original culture volume of permeabilized cells in 20 mM Tris–HCl buffer at pH 7.5. The procedure could be adapted to cultures in a large volume (1 l), medium volume (10 ml) and a small volume (200 µl) as detailed in “Methods” section.

**Fig. 2:**
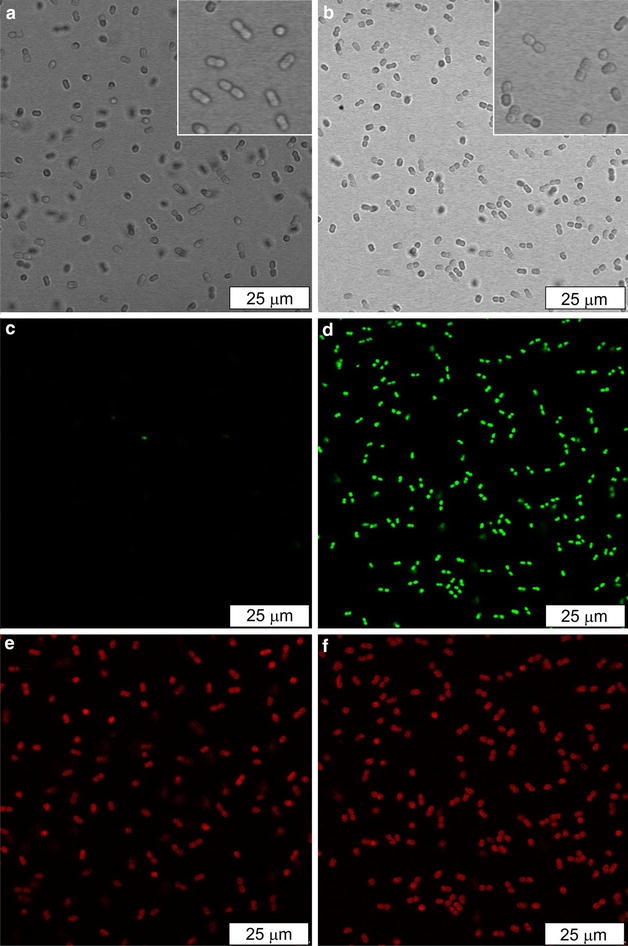
Microscopic analysis of the *Synechococcus* sp. PCC 7002 negative control and B-PER treated cells stained with the nucleic acid stain SYTOX Green. Bright field images of the negative control (**a**) and the permeabilized cells (**b**). Fluorescence imaging of SYTOX GREEN upon excitation at 488 nm and emission detection between 515 and 535 nm for the negative control (**c**) and the permeabilized cells (**d**). The fluorescence imaging of Chl *a* upon excitation at 573 nm and emission detection between 590 and 640 nm for the negative control (**e**) and the permeabilized cells (**f**). The same setting for imaging was applied for imaging of the control and the B-PER treated cells. At least three independent preparations were analyzed and representative images are shown here

Bright field images of the B-PER treated cells revealed the characteristic rod shape (Fig. 2b, insert), similar to the negative control cells (Fig. 2a, insert). Measurements of the lengths of the cells in the longitudinal (length) and transverse (width) orientations revealed that the length-to-width ratios in the B-PER-treated cells and the negative control cells were 1.43 ± 0.24 and 1.51 ± 0.2, respectively (*N* = 12 for each sample, *P* > 0.05). Therefore, the permeabilized cells possessed the overall cell morphology of the negative control cells. The B-PER treated cells were found to contain the similar amount (96 ± 1.2%) of Chl *a* as observed for the negative control cells (*N* = 3, *P* < 0.05), hence the treatment causes only a minor loss of Chl *a*.

SYTOX GREEN is a widely used dye in cell viability tests. It is impermeant to cells with intact membranes, whereas it can diffuse through permeabilized membranes and exhibit 500-fold enhancement in fluorescence intensity at around 530 nm upon binding with nucleic acids inside the cells. Confocal scanning laser microscopy revealed that, after treatment with SYTOX GREEN, the B-PER treated cells gave rise to a strong fluorescence signal at 530 nm (Fig. 2d) that coincided with the Chl *a* autofluorescence (Fig. 2f). On the other hand, the negative control cells did not give rise to a notable level of fluorescence at 530 nm after the SYTOX GREEN treatment (Fig. 2c), whereas the Chl *a* autofluorescence was clearly visible (Fig. 2e). Taken together these results indicate that B-PER treatment permeabilizes the *Synechococcus* 7002 cells.

### YFP and cellular proteins are retained in the permeabilized cells during the B-PER treatments

**Fig. 3:**
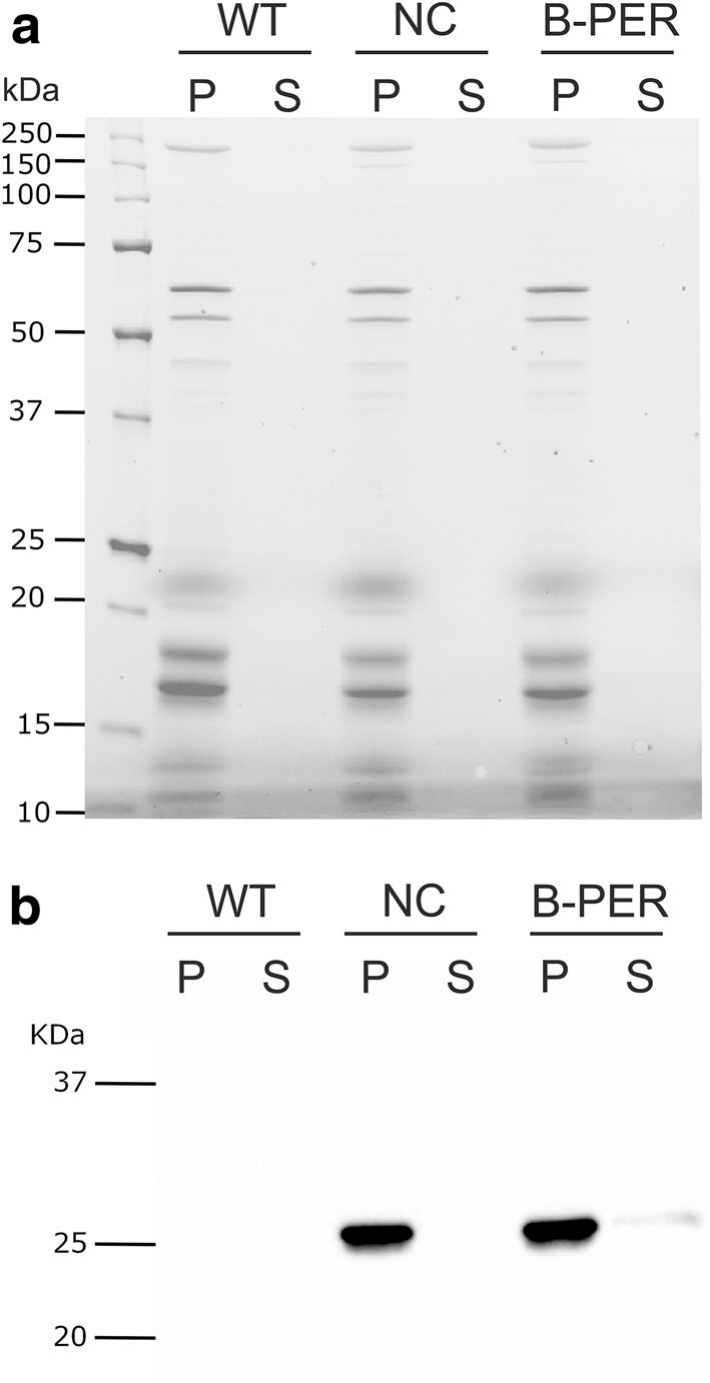
Immunoblot analysis of YFP expressed in the *Synechocystis* 6803 (YFP-mut strain) negative control and B-PER treated cells. **a** Total proteins separated by SDS-PAGE and visualized upon the UV illumination. **b** Immunoblot of YFP probed with anti-GFP polyclonal antibody. NC, the negative control cells; B-PER, the B-PER treated cells; P, pellets; S, supernatant

A quantitative assay was performed to assess the amount of the intracellular proteins lost during the B-PER treatment in order to probe the degree of cell lysis. To this end, a strain of *Synechocystis* 6803 expressing YFP in the cytoplasm (YFP-mut) was used. As described below, *Synechocystis* 6803 cells were also permeabilized by the B-PER treatment. The cells were treated with the buffer and the B-PER reagent and the supernatants and the cell pellets obtained after the centrifugation were analyzed for the presence of total proteins and YFP. When the total proteins in the protein gels were visualized under the UV illumination, most of the cellular proteins were found in the pellets regardless of the sizes, at least between 10 and 250 kDa (Fig. 3a). When the presence of YFP was detected by immunoblotting, most YFP was found in the pellets while a small fraction, accounting for up to 8% of the total YFP based on a densitometry analysis of the blot, was detected in the supernatants (Fig. 3b). These results indicate that only a small fraction of the cells were lysed during the B-PER treatment, and most intercellular proteins remained inside the permeabilized cells.

### Enzyme activity assays using the permeabilized cells of *Synechococcus* 7002 and *Synechocystis* 6803

**Fig. 4:**
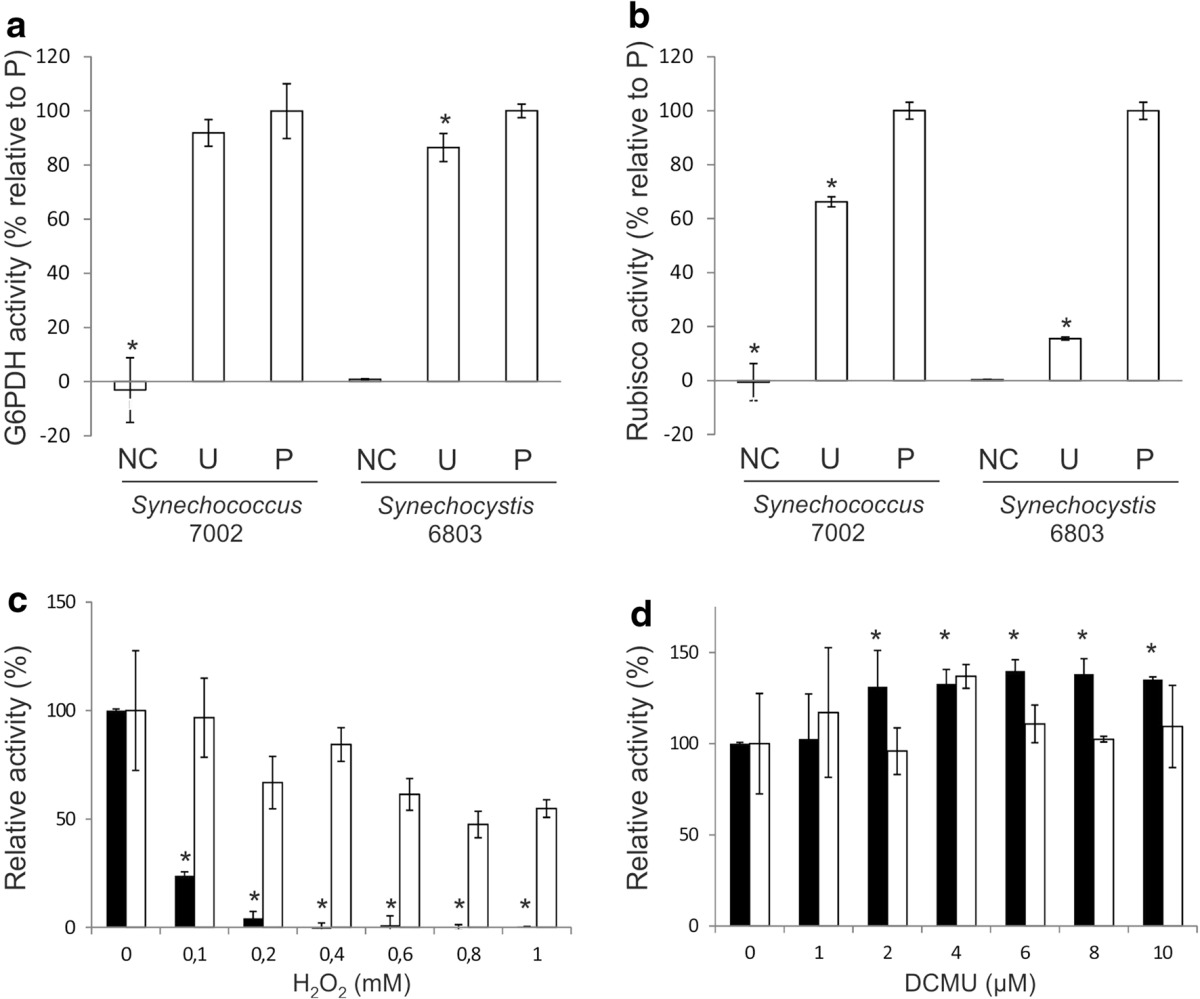
The G6PDH and Rubisco enzyme activities measured for the control and permeabilized *Synechococcus* 7002 and *Synechocystis* 6803 cells. **a** The G6PDH activity. **b** The Rubisco activity. NC, U, and P indicate the negative control cells, the cell-free lysates obtained by ultrasonication, and the permeabilized cells, respectively. The activity measured for each sample was normalized to the Chl *a* content of the same sample, and then expressed as relative (%) to the activity detected for the permeabilized cells (P) of the same species. For each species, the same starting cell materials were used for the different treatments and six independent samples were analyzed for each treatment. **c** and **d** The relative activities of G6PDH (*black bars*) and Rubisco (*white bars*) in *Synechococcus* 7002 cells treated with various concentrations of **c** H_2_O_2_ and **d** DCMU for 2 h prior to permeabilization. The activities are expressed as relative to the activity measured for cells that have been incubated with water as the negative control. At each concentration, three independent samples were analyzed. Student’s *T* test was performed and statistically significant differences (*P* < 0.05) in comparison to the negative control are marked by *asterisks*

The permeabilized *Synechococcus* 7002 cells were applied directly to the G6PDH activity assay using exogenously supplied substrates (G6P and NADPH). The assay was performed in a 96-well plate and the kinetics at 340 nm was monitored using a plate reader, enabling simultaneous analysis of a large number of samples. As expected, the negative control cells did not show activity above the detection limit (Fig. 4a), because of the impermeable membrane barrier in the intact cells. In contrast, a pronounced activity, 0.91 nmol ± 0.091 NADH min^−1^ µg^−1^ Chl *a* (*N* = 6), was detected for the permeabilized cells (Fig. 4a). For comparison, cell-free lysates were prepared from the same cultures and the G6PDH activity was determined. The activity detected for the cell-free lysates was comparable to that observed for the permeabilized cells (*N* = 6, *P* > 0.05) (Fig. 4a). It should be noted that additional control assays, wherein G6P was omitted, gave rise to the baseline level of activity (~0 nmol ± 0.091 NADH min^−1^ µg^−1^ Chl *a*), which verified that the detected activities are due to G6PDH. Taken together, these results indicate that the B-PER treatment is effective in permeabilizing *Synechococcus* 7002 cells. The permeabilization protocol was applied to *Synechocystis* 6803 and the G6PDH activity was determined. Again, no activity was detectable for the negative control cells, whereas a notable activity, 1.8 nmol ± 0.045 NADPH min^−1^ µg^−1^ Chl *a* (*N* = 6), was detected for the B-PER treated *Synechocystis* 6803 cells, indicating that the B-PER treatment also permeabilized *Synechocystis* 6803. Cell-free lysates prepared from the same culture of *Synechocystis* 6803 showed a comparable but slightly lower level of the G6PDH activity to the permeabilized cells (Fig. 4a), suggesting that the B-PER treatment is quite efficient.

Next, the Rubisco activity was determined for the permeabilized and the negative control cells using a label-free enzyme-coupled assay by monitoring the kinetics of the NADH oxidation. While no activity was detectable for the negative control cells of *Synechococcus* 7002 and *Synechocystis* 6803, notable activities, 4.0 ± 0.11 and 5.2 ± 0.17 nmol NAD^+^ min^−1^ µg^−1^ Chl *a*, respectively, were measured for the permeabilized cells (Fig. 4b). The activities were also detected for the cell-free lysates; however they were significantly lower than those of the permeabilized cells (Fig. 4b). It is plausible that the ultrasonication compromises the integrity of carboxysomes, hence the availability of CO_2_ in the vicinity of Rubisco, or that the subsequent centrifugation might have caused precipitation of a fraction of carboxysomes that were entrapped within the cell debris. It should be noted that additional control assays, wherein RuBP was omitted, gave rise to the baseline level of activities (~0.1 nmol NAD^+^ min^−1^ µg^−1^ Chl *a*), which verified that the detected activities were due to Rubisco.

Lastly, to test if the protocol can be adapted to analyze the activities of G6PDH and Rubisco upon environmental stimuli, the whole cells of *Synechococcus* 7002 were treated with selected chemicals for 2 h and were subsequently permeabilized for the activity assay of G6PDH and Rubisco. In order to increase the throughput of analysis, the treatments of the cultures were performed in 96-well plates wherein cultures in individual wells were side-by-side incubated in the presence of varying concentrations of H_2_O_2_ or DCMU. These chemicals were chosen because they are readily taken up by the whole cells. H_2_O_2_ is a reactive oxygen species and naturally accumulates inside cyanobacterial cells at a relatively low concentration (ca. 50 µM) [53], while exogenous application of a higher concentration of H_2_O_2_ (≥0.35 mM) has been shown to inhibit the growth of *Synechocystis* 6803 [54]. DCMU is an herbicide that specifically binds Photosystem II and blocks the photosynthetic electron transport at Photosystem II, leading to an oxidized redox state of the plastoquinone pool without causing a major negative impact on growth at the concentrations used in this study [55]. The cultures were incubated with varying concentrations of H_2_O_2_ or DCMU up to 1 mM and 10 µM, respectively, for 2 h at a low light intensity (20 µmol photon m^−2^ s^−1^) and the cells were separated from the bulk of the chemicals by centrifugation prior to permeabilization. As shown in Fig. 4c, the G6PDH activity dramatically diminished in the presence of 0.1 mM H_2_O_2_, and virtually no activity was detectable above the detection limit in the presence of 0.4 mM or higher concentrations of H_2_O_2_. On the other hand, the Rubisco activity remained largely unaltered although the mean activities gradually declined. In contrast, the treatment with DCMU induced the G6PDH activity by up to 40% of the control level at the concentrations of 2 µM and higher, while the Rubisco activity remained largely unaffected (Fig. 4d). These results indicate that the cellular G6PHD activity is modulated by relatively low concentrations of H_2_O_2_ and DCMU, while the Rubisco activities remain largely unaffected under the conditions tested.

### Storage of the permeabilized cells

**Fig. 5:**
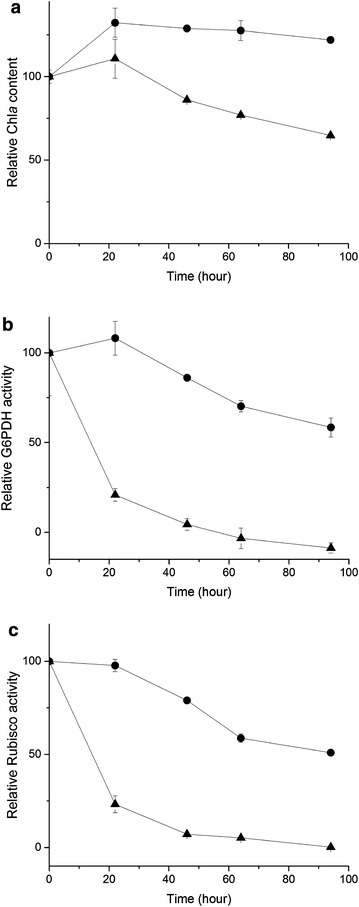
Impact of storage temperatures on the Chl *a* content and activities of G6PDH and Rubisco in the permeabilized cells of *Synechococcus* 7002. **a** the Chl *a* contents, **b** the G6PDH activity, and **c**, the Rubisco activity. The values were expressed as relative to the values at 0 h. *Circles* and *triangles* indicate cells stored at 4 °C and at RT (−25 °C), respectively. *Error bars* represent standard deviation based on three independent samples

The impact of storage was assessed on the Chl *a* content and the G6PDH and Rubisco activities in the permeabilized *Synechococcus* 7002 cells. The Chl *a* content during the storage at 4 °C was largely unaltered during the course of the measurements up to 94 h (Fig. 5a). The G6PDH and Rubisco activities were nearly unchanged within the first 22 h of incubation at 4 °C, and gradual reductions in the activities followed (Fig. 5b, c). By 94 h, the G6PDH and Rubisco activities declined to ca. 58 and 50% of the initial levels, respectively (Fig. 5b, c). In contrast, the storage at the room temperature caused a slight reduction in the Chl *a* content by 22 h and by 96 h up to 35% of the initial value disappeared (Fig. 5a). Under this condition, the enzyme activities declined dramatically; 80 and 77% of the G6PDH and Rubisco activities, respectively, disappeared by 22 h and no activity was detectable above the base line by 96 h (Fig. 5b, c).

**Fig. 6:**
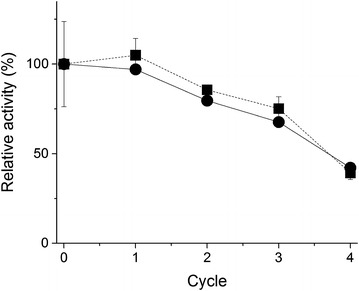
Impact of storage at −20 °C and cycles of freezing and thawing. *Squares* with a *solid line* and *circles* with a *dotted line* indicate the G6PDH and Rubisco relative activities, respectively. The activities are expressed as relative to those detected for the untreated samples. The results of the permeabilized cells by B-PER are shown. Three independent samples were analyzed for each cycle

It is often desirable that the samples can be kept at −20 °C for later analysis. Therefore, the permeabilized *Synechococcus* 7002 cells were subjected to cycles of freezing at −20 °C and thawing, and the G6PDH and Rubisco activities were determined after each cycle. The activities were unaltered after the first cycle of freezing and thawing in comparison to the untreated samples, while further cycles caused the activities to decline (Fig. 6).

## Discussion

The present study shows that the treatment by the B-PER reagent successfully permeabilized *Synechococcus* 7002 and *Synechocystis* 6803 cells without causing significant lysis of the cells. The permeabilized cells maintained most of the detectable intracellular proteins including YFP, and could be directly applied for determining the G6PDH and Rubisco activities using exogenously provided substrates and coupling enzymes. The enzymatic activities measured for the permeabilized cells were comparable to or greater than those measured for cell-free lysates that have been prepared by ultrasonication. These results indicate that the permeabilization achieved in the current study is highly efficient.

The G6PDH activity detected in this study was ca. 0.9 nmol NADPH min^−1^ µg Chl *a* ^−1^ for *Synechococcus* 7002 and ca. 1.8 nmol NADPH µg Chl *a* ^−1^ min^−1^ for *Synechocystis* 6803. Previously Summers et al. have reported the G6PDH activity of between 30 and 60 nmol NADPH mg protein^−1^ min^−1^ for cell lysates obtained from *Nostoc punctiforme* sp. ATCC 29133 [14]. Taking into account that typical *Synechococcus* 7002 and *Synechocystis* 6803 cells contain approximately 3 µg Chl *a* OD_730_ nm^−1^ cm^−1^ and approximately 90 µg proteins OD_730_ nm^−1^ cm^−1^ under the normal growth conditions as employed in this study [56–58], the activities obtained in this study can be estimated as 30 and 60 nmol NADPH mg protein^−1^ min^−1^ for *Synechococcus* 7002 and *Synechocystis* 6803, respectively, which is consistent with the previous report. The Rubisco activities detected in this study were 4 nmol NADH µg Chl *a* ^−1^ min^−1^ for *Synechococcus* 7002 and 5.2 nmol NADH µg Chl *a* ^−1^ min^−1^ for *Synechocystis* 6803, which are equivalent of 2 and 2.6 nmol CO_2_ fixed µg Chl *a* ^−1^ min^−1^, respectively. These values are largely consistent with the cellular Rubisco activity of approximately 1.5 nmol CO_2_ fixed µg Chl *a* ^−1^ min^−1^ as previously reported for the permeabilized *Synechococcus* sp. PCC 7942 following the incorporation of ^14^C-labeled CO_2_ into the acid precipitated biomass [37]. Hence the activities measured by using the permeabilized cells and exogenous addition of substrates and coupling enzymes, in the case of the Rubisco assay, are in agreement with the previously reported activities of these enzymes in cyanobacteria.

The permeabilization protocol was adapted to cells that were pre-treated with chemicals (H_2_O_2_ and DCMU) in 96-well plates. This approach significantly facilitated the throughput of analysis and was particularly useful for assessing the impacts of different concentrations of the chemicals on the cellular enzyme activities under otherwise the identical conditions. Notably the G6PDH activity declined substantially after the 2-h incubation in the presence of 0.1 mM H_2_O_2_, while its activity level increased up to 40% of the control value in the presence of 2 µM DCMU. The result obtained upon treatment with DCMU was expected, because the inhibition of the photosynthetic electron transport chain by DCMU would decrease the size of the reduced thioredoxin pool, which would lead to activation of G6PDH [11–16]. The altered redox status of the thioredoxin pool may be also expected to induce changes in the abundance of Rubisco. For instance, the previous in vitro studies have shown that the oxidizing agents retard translation of Rubisco large subunit and impair the stability of the protein complex [17, 19, 20]. However, upon the DCMU treatments, no significant change in the activity of Rubisco was observed under the conditions tested. It is possible that the redox modulation of Rubisco in vivo might be more subtle than in vitro, that a more dramatic change in the redox status is needed to induce changes in the abundance of active Rubisco, or that other cellular factors play a role in regulating the redox modulation of Rubisco. Concerning the effect of H_2_O_2_, underlining physiological mechanisms leading to the observed changes in the G6PDH activity is unclear. Previously a short-term incubation (up to 10 min) of spinach chloroplast lysates with a high concentration (0.75 mM) of H_2_O_2_ has been reported to elevate the G6PDH activity [13], whereas in the cell lysate of the cyanobacterium *Anabaena* sp. PCC 7120 incubation in the presence of oxidized thioredoxins did not elevate the G6PDH activity [12]. Hence the results obtained in this study may represent adaptive responses as a result of the treatments, rather than direct redox-mediated modulation of the G6PDH activity.

## Conclusions

An easy and efficient method for permeabilizing cyanobacterial cells has been established. The procedure is scalable with respect to volumes and readily adaptable to 96-well plates as well as a larger (1 l) culture volume. The permeabilized cells can be directly applied to isotope label free assays of G6PDH and Rubisco, generating results that are consistent with the previous reports. The procedure takes less than 30 min and can process multiple samples (e.g., in 96-well plates) at a time. Finally, the prepared samples can be stored at −20 °C. The protocol presented here may be extended to studies of a collection of natural isolates, mutants and engineered strains, other cyanobacterial species, and also analysis of other cellular enzymes.

## Abbreviations

Chl *a*: chlorophyll *a*
G6P: glucose-6-phosphate
G6PDH: glucose-6-phosphate dehydrogenase
Rubisco: ribulose-1,5-bisphosphate carboxylase/oxygenase
YFP: yellow fluorescent protein
